# Supplementary material for: Effectiveness and Safety of Ayurvedic Medicines in Type 2 Diabetes Mellitus Management: A Systematic Review and Meta-Analysis
Source: Front Pharmacol. 2022 Jun 8;13:821810. doi: 10.3389/fphar.2022.821810 (PMC9213670; doi:10.3389/fphar.2022.821810)
Supplement: Supplementary file 1 [file DataSheet1.zip › Supplementary Material/Forest and Funnel Plots/Tinospora cordifolia/TG.pdf]

Tinospora cordifolia - TG

Study	Intervention N	Intervention Mean	Intervention SD	Comparator N	Comparator Mean	Comparator SD	Mean Diff. with 95% CI	Weight (%)
Balasubramaniam 2010	20	122.00	15.00	20	125.00	13.00	-3.00 [-11.70, 5.70]	88.37
Roy 2015	29	124.30	45.80	30	129.90	48.10	-5.60 [-29.58, 18.38]	11.63
**Overall**							-3.30 [-11.48, 4.88]	

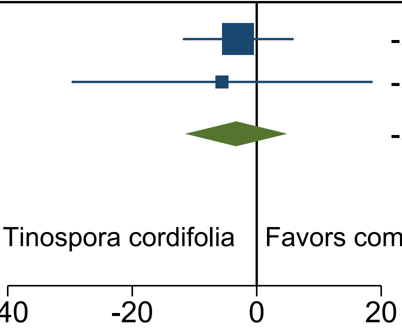

Heterogeneity: $\tau^2 = 0.00$, $I^2 = 0.00\%$, $H^2 = 1.00$

Test of $\theta_i = \theta_j$: Q(1) = 0.04, p = 0.84

Test of $\theta = 0$: z = -0.79, p = 0.43

Random-effects REML model
